# Liquid Biopsy in Lung Cancer: Clinical Applications of Circulating Biomarkers (CTCs and ctDNA)

**DOI:** 10.3390/mi9030100

**Published:** 2018-02-28

**Authors:** Minji Lim, Chi-Ju Kim, Vijaya Sunkara, Mi-Hyun Kim, Yoon-Kyoung Cho

**Affiliations:** 1 Department of Biomedical Engineering, School of Life Sciences, Ulsan National Institute of Science and Technology (UNIST), UNIST-gil 50, Ulsan 44919, Korea; ming4279@unist.ac.kr (M.L.); chijukim1@gmail.com (C.-J.K.); chidralavijaya@gmail.com (V.S.); 2 Center for Soft and Living Matter, Institute for Basic Science (IBS), UNIST-gil 50, Ulsan 44919, Korea; 3 Department of Internal Medicine, Pusan National University School of Medicine and Biomedical Research Institute, Pusan National University Hospital, 179, Gudeok-ro, Seo-Gu, Busan 49241, Korea; mihyunkim@pusan.ac.kr

**Keywords:** liquid biopsy, circulating biomarkers, circulating tumor cells, circulating tumor DNA, non-small cell lung cancer

## Abstract

Lung cancer is by far the leading cause of cancer death worldwide, with non-small cell lung cancer (NSCLC) accounting for the majority of cases. Recent advances in the understanding of the biology of tumors and in highly sensitive detection technologies for molecular analysis offer targeted therapies, such as epidermal growth factor receptor (EGFR) tyrosine kinase inhibitors. However, our understanding of an individual patient’s lung cancer is often limited by tumor accessibility because of the high risk and invasive nature of current tissue biopsy procedures. “Liquid biopsy”, the analysis of circulating biomarkers from peripheral blood, such as circulating tumor cells (CTCs) and circulating tumor DNA (ctDNA), offers a new source of cancer-derived materials that may reflect the status of the disease better and thereby contribute to more personalized treatment. In this review, we examined the clinical significance and uniqueness of CTCs and ctDNA from NSCLC patients, isolation and detection methods developed to analyze each type of circulating biomarker, and examples of clinical studies of potential applications for early diagnosis, prognosis, treatment monitoring, and prediction of resistance to therapy. We also discuss challenges that remain to be addressed before such tools are implemented for routine use in clinical settings.

## 1. Introduction

Lung cancer is the most common cause of cancer-related mortality in both men and women worldwide, with 1.8 million new cases reported every year [1,2]. In contrast to the increase in the survival rate for most other cancers, the 5-year relative survival rate of lung cancer remains at 18%. In the USA alone, lung cancer was responsible for an estimated 158,040 deaths in 2017, which was approximately 26% of all cancer-related deaths in that year. The high mortality rate of lung cancer is partly the result of more than half of all cases being diagnosed at an advanced stage, at which the five-year survival rate is only 4% [1]. It is important to expand the X-ray pre-screening for general population to detect cancer in earlier stages. In addition, it is critical to develop more sensitive technologies for early detection of lung cancer and to improve lung cancer survival rates.

Non-small cell lung cancer (NSCLC) represents 85% of all lung cancers, with epidermal growth factor receptor (EGFR) mutations occurring in 10–30% of NSCLC patients. Recent advances in our understanding of molecular abnormalities in lung cancer and in highly sensitive molecular analysis technologies have revealed that targeted therapies such as the use of EGFR tyrosine kinase inhibitor (TKI) drugs can deliver improved clinical outcomes for certain groups of patients with advanced NSCLC [3,4,5,6,7]. Currently, tissue biopsy is conducted for most patients with NSCLC to obtain molecular information about their tumors. However, the efficacy of targeted therapy is limited because of the almost inevitable development of resistance. The invasive nature of tissue biopsy is an obstacle to the frequent sampling that would make it possible to better understand tumor dynamics and drug response. In addition, local sampling by tissue biopsy can be biased because of the heterogeneity of tumors and failure to detect the occurrence of metastasis at distant sites.

Less invasive “liquid biopsy” can provide insight into the real-time dynamics of lung cancer via more frequent analysis of circulating biomarkers, such as circulating tumor cells (CTCs) and circulating tumor DNA (ctDNA). Furthermore, liquid biopsy is believed to offer a more comprehensive picture of the disease, because markers circulating in blood may contain cancer-associated materials from multiple disease sites in the body (Table 1). However, the extreme rarity of tumor-associated biomarkers in blood has been a great challenge to achieving the goals desired of liquid biopsy. Recent innovations in microfluidic chip-based ultrasensitive rare cell isolation technologies and affordable molecular detection technologies, such as next-generation sequencing (NGS) and droplet digital polymerase chain reaction (ddPCR), have produced promising clinical test results, which suggest that liquid biopsy-based personalized medicine will become a reality in the near future.

While CTCs have still not been validated for use in clinical settings for disease management for patients with NSCLC, ctDNA was approved by the U.S. Food and Drug Administration (FDA) in 2016 as the first liquid biopsy test for patients with NSCLC in which ctDNA is analyzed for the presence of specific mutations to identify patients eligible for treatment with EGFR-targeted therapy [8]. In this review, we present a brief overview of clinical perspectives concerning and opportunities for the use of liquid biopsy in lung cancer, focusing specifically on the following: identification of actionable mutations, such as sensitizing (19 del and L858R) and resistant (T790M) EGFR mutations; the significance and uniqueness of the two most popular circulating biomarkers, i.e., CTCs and ctDNA; and the detection methods for each biomarker and the current limitations of these methods. We also discuss the key areas of potential clinical applications of liquid biopsy using CTCs and ctDNA in early diagnosis, prognosis, and monitoring of response and resistance to treatment, as well as obstacles that remain to be overcome before liquid biopsy is implemented routinely in clinical settings.

## 2. EGFR Mutations and Resistance Mechanisms

**Figure 1:**
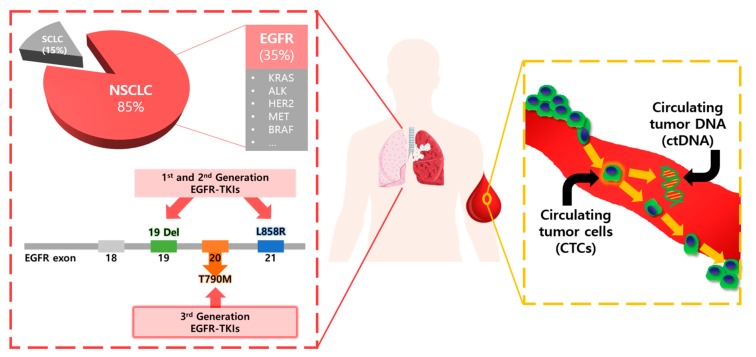
Schematic illustration of clinical utility of liquid biopsy for lung cancer. Circulating biomarkers (CTCs and ctDNA) are used for the identification of actionable mutations, such as sensitizing (19 del and L858R) and resistant (T790M) EGFR mutations.

EGFR TKI drugs, such as gefitinib, erlotinib and afatinib are used as the first-line therapy for patients with advanced NSCLCs [3,4,5,6,7]. As schematically shown in Figure 1, some groups with specific activating mutations, deletions in exon 19 (19 Del), and substitutions in exon 21 (L858R) in the tyrosine kinase domain of EGFR show dramatic response to EGFR TKIs. A large portion of the responding groups are Asian women with adenocarcinoma with little or no previous history of smoking. This suggests a new paradigm of personalized medicine in which the effectiveness of a targeted drug could be predicted for specific patients by mutation analysis.

However, EGFR inhibitors often work only for several months or more; eventually, the drugs stop working because of the development of resistance. One of the major resistance mechanisms is a secondary mutation in EGFR (T790M), which is found in 48% to 62% of EGFR TKI-resistant patients. Recently, a third-generation EGFR TKI, osimertinib, was approved by the FDA for T790M positive patients and has shown significantly better efficacy than platinum-based therapy. Therefore, early detection of T790M mutation during the first-line therapy and changing of the drug to 3^rd^ generation EGFR TKIs included osimertinib are extremely important in disease management for NSCLC patients with EGFR mutations [9].

## 3. Significance and Uniqueness of CTCs and ctDNA as Liquid Biopsy Biomarkers

### 3.1. CTCs

CTCs are tumor cells that are disseminated from primary tumors and/or metastatic lesions and circulate in the blood stream. CTCs are distinguished from other blood cells by their positive expression of epithelial markers, including epithelial cell adhesion molecules (EpCAM) and cytokeratins (CK) and negative expression of the white blood cell-specific marker CD45 [10]. During intravasation into blood vessels, some CTCs undergo epithelial-to-mesenchymal transition (EMT), and during extravasation into secondary organs, it undergoes mesenchymal-to-epithelial transition (MET). Thus, CTCs in the blood stream have heterogeneous levels of biomarker expression related to EMT and MET [11]. Heterogeneous CTCs can be divided into various subgroups: intact single CTCs, apoptotic CTCs, and CTC clusters. The size range of CTCs (8–20 µm) is slightly greater than that of other blood cells, but the size is also heterogeneous and can be similar or smaller than surrounding WBCs [12,13].

As shown in Table 2, CTCs contain various cellular and subcellular components that can be used for further downstream analysis; e.g., intact DNA for well-known mutation analysis and novel marker discovery, RNA for gene expression profiling, and various biomarkers for proteomic analysis. Direct visualization of cellular morphology for the identification of malignant phenotypes is an important and unique aspect of CTCs in comparison to other circulating biomarkers. In addition, immunocytochemistry (ICC) [14] and fluorescent in situ hybridization (FISH) [15,16] can be performed on CTCs to identify their phenotype and detect some genomic alteration. CTCs can be divided into various subgroups, according to the cell diameter or nuclear fraction, and each subgroup is analyzed for its characteristics [17]. Isolated CTCs are cultured in vitro to establish the permanent CTC cell line for further studies. Furthermore, they can be used in various in vivo, ex vivo, and in vitro experiments in functional studies. CTC-derived explants (CDXs), patient-derived xenograft models (PDXs), and ex vivo-cultured CTCs can be used in drug screening, which can contribute to personalized therapy [18,19,20].

However, CTCs are extremely rare in comparison to other types of blood cells (1–10 CTCs/10^6^ blood cells in 1 mL of blood) [21]. Furthermore, CTCs are heterogeneous, both in terms of surface protein markers and physical characteristics. Therefore, it is a great challenge to isolate CTCs with high sensitivity and specificity. For example, CTCs isolation technologies that use anti-EpCAM antibodies cannot isolate cells that have undergone EMT or have low EpCAM expression. Even if 99.9% of blood cells are purified, the purity of the isolated CTCs is below 1%, which is at the detection limit of the currently available molecular analysis techniques. Thus, extremely sensitive and specific isolation and detection methods are required to improve the clinical utility of liquid biopsy. While the CellSearch technology has been approved by the FDA for breast, colorectal, and prostate cancer testing, the use of CTCs for lung cancer testing is still under study.

### 3.2. ctDNA

Tumor-related genetic alterations can be detected in ctDNA, which is fragmented DNA freely circulating in the blood stream that is shed from tumor tissue by apoptosis, necrosis, and secretions from tumor cells [22]. The short length of DNA (~180 base pairs) is reminiscent of the ctDNA generated by apoptotic degradation of cellular DNA [23,24]. Because of the short length of ctDNA, its clearance is very rapid, with a half-life of less than 2 h [25]. For ctDNA analysis, plasma samples are preferable to serum samples to avoid possible contamination of wild-type DNA released from other blood cells. The unfavorable stability of ctDNA is advantageous for real-time monitoring of the disease status, although it adversely affects the sensitivity of the detection. Furthermore, ctDNA can originate from any tumor lesion in the body, so sampling bias is minimized. Thus, ctDNA offers an advantage in drug response monitoring of providing crucial evidence concerning whether a tumor has recently developed resistance or not (Table 2). The cobas^®^ EGFR Mutation Test v2, which is a PCR-based liquid biopsy test for NSCLC, has recently been approved by the FDA. However, the extremely low fraction of tumor-specific genetic alterations in a “sea” of normal cell-free genomic DNA requires further development of highly sensitive, advanced molecular detection technologies, as well as ctDNA-specific isolation methods. Recent advances in personalized cancer management have been expedited, and significant progress has been made in ultrasensitive molecular characterization methods, such as NGS and third-generation PCR technologies (e.g., ddPCR) [26,27,28].

In summary, both CTC and ctDNA-based liquid biopsy can contribute to cancer diagnosis, prognosis, and personalized treatment. Detection of EGFR mutation by means of ctDNA analysis is an exciting application of liquid biopsy and has been recently approved by the FDA. CTCs offer advantages in detection of mutation, analysis of multiple biomarkers (DNA, RNA, proteins), and morphological and functional analysis of intact cancer cells. Liquid biopsy using multiple types of biomarkers may minimize sampling bias and provide a more comprehensive picture of disease status. For example, ctDNA-based detection may be less vulnerable to the isolation methodology and may better represent cancer heterogeneity and be more useful in longitudinal surveillance of cancer therapy. The lack of materials and multiple choices for isolation technologies remain unsolved issues associated with CTC analysis. On the other hand, cytochemistry and morphological analysis of intact cancer cells provides valuable information that is relevant to the established cytological criteria used in tissue biopsy. Furthermore, in vivo functional assays using a PDX model developed from CTC analysis results can provide fundamental knowledge of the metastatic process and disease progression. However, both biomarkers are rare and fragile and require more sensitive techniques for isolation from other blood components with high yield and high purity. Therefore, the improvement of the sensitivity specificity of the analysis of target biomarkers has been an active research area in recent decades, both in chip-based rare cell isolation and advanced molecular characterization. In the remainder of this review, we summarize representative commercialized products for liquid biopsy and related clinical reports on the prognosis for NSCLC patients. We also present some findings from recent studies on molecular diagnosis based on analysis of CTCs and ctDNA, and we present perspectives on the future of liquid biopsy for patients with NSCLC.

## 4. Technologies for CTC Analysis and Clinical Applications for Patients with NSCLC

### 4.1. Prognosis for Patients with NSCLC Using Commercially Available CTCs Isolation Devices

CellSearch (J&J, Veridex) is the only FDA-approved method for enumerating CTCs. The separation mechanism is based on the isolation of CTCs using magnetic beads conjugated with EpCAM antibody. Although CellSearch is approved for use in assessing the prognosis of patients with metastatic breast cancer (2004), colorectal cancer (2007), and prostate cancer (2008), it shows limited detection sensitivity for low EpCAM-expressed CTCs. It has not yet been approved for lung cancer, despite many studies that have been conducted to attempt to establish CTCs counts as diagnostic and prognostic tools for NSCLC patients. As shown in Table 3, several studies have shown that CTC counts may be useful as prognostic markers for patients with advanced lung cancer, even when the detection rate is relatively low. Most of these studies sought to identify baseline CTC counts for use as prognostic markers and reported significantly poor progression-free survival (PFS) or overall survival (OS) in patient groups with high CTC counts [29,30,31,32,33,34,35,36].

The first representative study of NSCLC patients was reported in 2011 and addressed the clinical meaning of baseline CTC counts measured by CellSearch. A total of 101 patients with advanced NSCLC (stage IIIA–IV) were divided into two groups according to their baseline CTC counts, with a cut off of 5 CTCs/7.5 mL between the two groups. In this study, both PFS and OS were significantly poorer in the CTC-positive group than in CTC negative group (median PFS: 6.8 months vs. 2.4 months, median OS: 8.1 months vs. 4.3 months). Moreover, patients who had less than 5 CTCs/7.5 mL at two sequential time points achieved much longer PFS and OS (median PFS: 7.6 months vs. 2.4 months, median OS: 8.8 months vs. 4.3 months) [29].

Other papers have reported the clinical importance of not only baseline CTC counts but also CTC counts over the course of treatment [30]. Among 37 evaluable advanced NSCLC patients’ samples, 75.7% of patients had positive baseline CTC counts (≥1 CTCs/7.5 mL), and a strong association was observed between baseline CTC counts and responses to treatment as measured by Response Evaluation Criteria in Solid Tumors (RECIST). More importantly, the changes observed in CTC counts 56 days after treatment were much more strongly correlated to survival than changes in CTC counts at 14 or 28 days after treatment (*p* value at 56 days: 0.006 vs. at 14/28 days: 0.104) [30]. These data suggest a correlation between decreases in CTC counts after treatment and longer PFS, which may indicate an early response to the therapy.

However, another study conducted with 59 advanced NSCLC patients showed that CTC counts were poorly correlated to the treatment response, although they were a good indicator of poor prognosis and the presence of distant metastasis [31]. Patients with CTC counts above the cutoff value of 2 CTCs/7.5 mL had significantly poor PFS and OS (median PFS: 6.2 months vs. 4.3 months, median OS: 11.2 months vs. 8.3 months). In addition, CTC counts 2 months after treatment were also well correlated with OS (*p* value of OS at baseline: 0.006 and at 2 months after: 0.008) [31].

The prognostic value of CTC subgroups has also been analyzed on the basis of characterization of cell morphology and the expression levels of specific biomarkers. In one study, among 43 patients with advanced NSCLC, those who had more than five morphologically intact CTCs showed significantly poor PFS and OS (median PFS: 7.6 months vs. 4.1 months, median OS: 10.7 months vs. 4.6 months) [32]. Furthermore, patients with an increase in intact CTCs after one cycle of chemotherapy had poorer PFS. This study involved testing of not only intact CTCs that met the calling criteria of the CellSearch system but also CTC-like objects, such as apoptotic CTCs and CK+ fragments. Interestingly, an apoptotic CTC-positive group (≥2 apoptotic CTCs/7.5 mL) also had poor PFS and OS (median PFS: 7.6 months vs. 3.4 months, *p* = 0.017; median OS: 10.5 months vs. 3.6 months, *p* = 0.001) [32].

A recent study of 125 patients with advanced and metastatic NSCLC showed that total CTC counts (≥5 CTCs/7.5 mL) can be used as a prognostic biomarker for OS (HR 0.55, 95% CI 0.33–0.92, *p* = 0.022) but not PFS (HR 0.68, 95% CI 0.42–1.1, *p* = 0.118). When the CTCs counts of these patients were analyzed further based on Vimentin (Vim) expression and genetic subtypes (KRAS mutation, EGFR mutation, and ALK rearrangement), Vim-expressed CTCs (≥1 Vim (+) CTCs) were significantly higher in patients with EGFR-mutated cancer (total CTC (+) 57.1% vs. 30.1, *p* = 0.038; Vim-expressed CTC positive 42.9% vs. 15.1%, *p* = 0.013), whereas no significant prognostic improvement was obtained when the total CTC+ groups were subdivided into Vim(+) CTC and Vim(−) CTC groups. Vim(+) CTCs were reduced and not found in the subgroups of ALK-rearranged and KRAS-mutated subgroups, respectively. Because Vim is a protein marker that is related to EMT, it was suggested that EMT characteristics can vary depending upon the genetic subtypes of NSCLC patients. However, this analysis had an intrinsic limitation in that the analysis of Vim, which is a mesenchymal marker, was performed for the CTCs isolated by immunoaffinity capture using an epithelial marker, EpCAM antibody. Nonetheless, the results suggest the importance of genetic subtypes in the clinical utility of CTC analysis [33].

Although the majority of CTC analyses have been conducted using blood samples from patients with advanced NSCLC, it would certainly be a “holy grail” discovery if liquid biopsy could be shown to be used successfully in early screening of lung cancer. However, this has not yet been accomplished, although there have been a few reports of progress that are worthy of note [34]. For example, in one study, 30 pulmonary vein (PV) samples and 27 peripheral (Pe) blood samples from 30 NSCLC patients who were not in an advanced stage but rather an early stage (Stage I–IIIA) were collected to test the number of CTCs using CellSearch. Much higher numbers of CTCs were detected in the PV samples (43% of patients had 1–3093 CTCs/7.5 mL in the PV samples) than in the Pe samples (22% of patients had 1–4 CTCs/7.5 mL in the Pe samples). However, the CTC counts for the PV samples were not significantly correlated with survival. Only the CTC counts from the Pe samples were strongly correlated with poor disease-free survival (DFS) and OS (*p* = 0.011 and *p* = 0.037) [34].

To overcome the limitation of EpCAM-based isolation methods, separation techniques based on differences in physical properties have also been developed. For example, ISET (Rarecells) uses the size difference between CTCs (8–20 µm) and other blood cells (6–10 µm). Compared to immunoaffinity-based methods, size-based filtration is relatively simple and fast. Although the cell sizes are heterogeneous, the number of CTCs isolated in one study using a track-etched polycarbonate membrane with a pore diameter of 8 µm was higher than that isolated by CellSearch methods [35,36]. For example, in a CTC-positive group (based on a cutoff value of 50 CTCs/6 mL), 30.8% of 208 patients with NSCLC had significantly poorer PFS and OS than patients in a CTC-negative group (*p* = 0.002 and 0.001, [35]). In a follow-up paper, positive CTC detection was highly correlated with DFS (*p* < 0.0001) for 50% of patients, based on a cutoff value of 1 CTC/7.5 mL. Interestingly, positive CTC detection by means of both CellSearch and ISET was more strongly correlated to poor survival than was positive CTC detection by means of only one method [36].

The clinical meaning of CTCs counts remains controversial, although many studies have indicated that high CTCs count are strongly correlated to poor survival. CTC numbers vary widely, depending on the tumor types, the patient groups, and the isolation methods used. Further investigation is required to determine whether CTC counts can be used to predict the response to therapy. Additional characterization of CTCs using molecular or protein markers is required to improve the clinical usefulness of CTCs. These limitations will be overcome as more robust technologies with improved assay performance are developed. Many new microfluidic chip-based rare cell isolation technologies are capable of achieving higher yield and purity than the devices that are currently commercially available, although larger-scale clinical tests with various patient groups are required for the validation of CTCs as a prognostic marker in clinical studies.

### 4.2. Molecular Diagnosis of Lung Cancer Using CTCs Isolated by Microfluidic Chips

For CTC counts to be used for molecular diagnosis, the purity should be higher than the minimum required of downstream molecular analysis techniques, such as PCR and NGS. Therefore, many devices have been developed to improve the capture efficiency and purity. In this section, we review the most recently developed devices that have been used for molecular analysis of CTCs isolated from patients with NSCLC, as summarized in Table 4.

**Figure 2:**
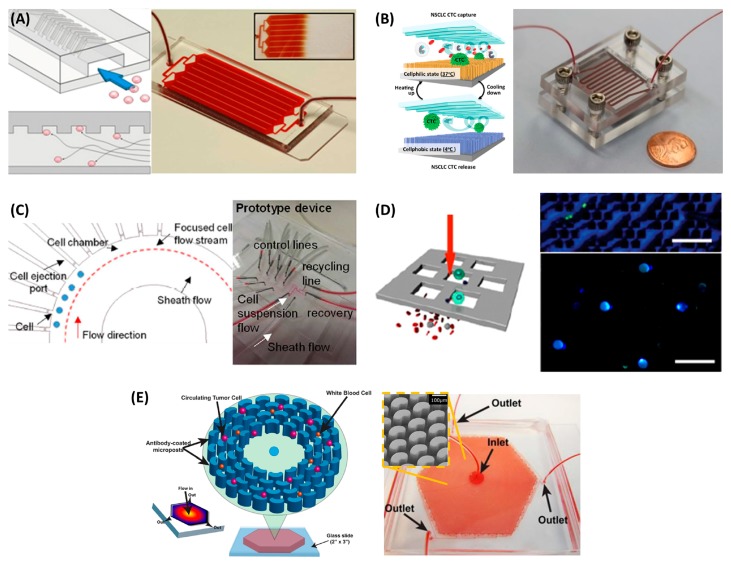
Examples of CTC isolation chips used in mutation analysis of CTCs from patients with NSCLC. (**A**) ^Hb^CTC-Chip, which has a herringbone pattern on the channel roof, was developed to improve capture efficiency (Right : 1″ × 3″ glass slide) [37,38]; (**B**) The purity of the cells captured by NanoVelcro chip, which has silicon nanowires on its bottom substrate to maximize the surface bound antibody, were further improved by thermoresponsive purification step [39]; (**C**) A microfluidic device (3.5 cm × 2.5 cm PDMS chip) capable of single-cell isolation and retrieval was developed to improve purity [40]; (**D**) A nanowell array filled with CTCs magnetically captured by a dense array of magnetic pores (MagSifter) were used for multigene expression profiling from individual CTCs (Upper scale bar: 200 µm, lower scale bar: 50 µm) [41,42]; (**E**) OncoBean Chip, which has bean-shaped posts coated with antibodies were developed to improve the throughput (Right: 2″ × 3″ glass slide) [43,44]. Reproduced from ref. [37,38,39,40,41,42,43,44,45] with permission from the United States National Academy of Sciences, American Chemical Society, Nature Publishing Group, Royal Society of Chemistry, and Wiley-VCH.

The ^Hb^CTC-Chip, which has a herringbone pattern on the channel roof, was developed to improve capture efficiency (Figure 2A). The contact frequency between CTCs and EpCAM antibody coated on the chip surface can be greatly enhanced by the microvortex formed as a result of the herringbone pattern. The ^Hb^CTC-Chip achieved a higher capture efficiency than a flat channel in a PC3 (a prostate cancer cell line) spiked experiment (capture efficiency of ^Hb^CTC-Chip: 79% ± 4.5% vs. flat chip: 29% ± 4.3%). In a clinical test with 15 metastatic prostate cancer patients, use of the ^Hb^CTC-Chip resulted in a detection rate of 93% (14/15 patients) [37]. The ^Hb^CTC-Chip has also been used to detect the T790M mutation in EGFR-mutant NSCLC patients and monitor the development of resistance to EGFR TKI therapy. In a study involving 40 patients with advanced NSCLC, genotyping was possible for 28 CTC samples (70%), and T790M could be detected for 14 (50%) patients, which was in 57% concordance with primary tissue biopsy results [38].

Another representative device used in immunoaffinity-based CTC isolation is the “NanoVelcro”, which has silicon nanowires on its bottom substrate, which results in a significantly enhanced surface area that can contain a larger amount of EpCAM antibody, and a micropattern on the roof that promotes efficient mixing. In a performance comparison with CellSearch, NanoVelcro achieved a much higher detection rate for 26 patients with prostate cancer; the detection rate of NanoVelcro was 76.9% (20/26 patients), while that for CellSearch was 30.8% (8/26) [45]. A follow-up study using an additional thermoresponsive purification step showed a significantly improved purity, from 35% to 98%, when the tests were performed with blood samples spiked with the H1975 NSCLC cell line (Figure 2B). In a study involving seven advanced NSCLC patients, 100% concordance in detection of the EGFR L858R mutation was achieved from CTCs and tissue biopsy samples. Two of the patients had the EGFR T790M mutation in relapse tissue samples, and this was also detected in patient-derived CTC samples [39].

To improve purity, a microfluidic device capable of single-cell isolation and retrieval was developed. The cells are first hydrodynamically focused in a single stream in the main fluidic channel and then guided into the single-cell capture chamber because of an intrinsic pressure difference (Figure 2C). A single trapped CTC can be released on demand to the main channel by positive pressure. Rare CTCs can be isolated with 100% purity. This microfluidic single-cell capture device was applied to the detection of EGFR L858R and T790M mutations from patient-derived CTCs by Sanger sequencing. A total of 26 CTCs were isolated from six of seven patients, which was in 100% concordance with the corresponding tissue biopsy samples [40].

Furthermore, multigene expression profiling from individual CTCs can be performed on a nanowell array filled with CTCs magnetically captured by a dense array of magnetic pores (MagSifter) [41]. Magnetic pore structure, Magsifter, was used to capture CTCs which plastered with anti-EpCAM magnetic nanoparticles. Magsifter had large equivalent magnetic forces at each pore and captured CTCs when external magnetic field was applied. When the external magnet was removed, captured CTCs can be released. The released CTCs seeded into the nanowell. A nanowell array can capture up to 25,600 cells, and single-cell-level multigene RT-PCR can be performed for the purpose of molecular characterization of single CTCs (Figure 2D). In one study, CTCs, defined as EpCAM+ cells with expression of both TERT and MET in a nanowell, were found in 31 of 35 (88.6%) patients, using a cutoff of 7 CTCs in 2 mL. The results also confirmed EGFR 19 del, L858R, and T790M mutations for seven NSCLC patients [42].

The OncoBean Chip, which has bean-shaped posts coated with a combination of multiple antibodies, such as EpCAM, EGFR, and CD133, with a radial flow enabling increased cell capture under high-flow-rate conditions, was developed to improve the throughput (Figure 2E). In a cell line-spiked experiment, the OncoBean Chip achieved more than 80% capture efficiency at a flow rate of 10 mL/h [43]. CTCs were detected in 69.4% and 83.3% in intraoperative Pe and PV blood samples, respectively, from a total of 36 patients with NSCLC. In this study, PV cases yielded much higher CTC counts (0–10,278/3 mL, with a median of 7.5 CTCs/3 mL) than Pe vein cases (0–28.5/3 mL, with a median of 1.3 CTCs/3 mL) [44]. In addition, CTC clusters were found in 50% of patients, and interestingly, the clusters found in PV samples were larger. Considering that the CTCs found in the PV samples were obtained before they had gone through systemic circulation, it can be inferred that PV is an enriched source of CTCs with larger-sized clusters. On the other hand, CTC clusters were either not found in the Pe samples or were smaller in size, which suggests that larger clusters may not able to enter systemic circulation or are dissociated in areas of smaller vasculature. It was found that the presence of the clusters in preoperative Pe samples was indicative of poor prognosis. Furthermore, mutations were found in five of six samples tested involving at least one of the genes CTNNB1, EGFR, KRAS, PIK3CA, and TP53. It is also noteworthy that gene expression analysis of CTCs recovered from the PV and Pe samples yielded different results. For example, TP53 expression was higher in CTCs from Pe samples, while ERCC1 was higher in PV samples. This may suggest molecular heterogeneity of CTCs from different sources.

In summary, there is evidence that isolated CTCs can be used as a biomarker to determine poor prognosis and to predict recurrence. To improve the sensitivity and specificity of isolated CTCs as a biomarker for cancer management, various microfluidic chips have been developed and used for molecular diagnostics in the selection of personalized treatment. A rather limited number of studies have been conducted to date involving patients with NSCLC, because of the rarity of CTCs in lung cancer. However, the results obtained to date are promising, and more advanced technologies are expected to be developed, because detection of mutation in subgroups of NSCLC patients is directly linked to the choice of actionable drugs and personalized cancer management.

## 5. Technologies for ctDNA Analysis and Clinical Applications for Patients with NSCLC

### 5.1. Highly Sensitive Detection Methods for ctDNA Analysis

Recent breakthroughs in sequencing technologies, especially in NGS, have expedited progress in the liquid biopsy field with respect to the use of ctDNA as a potential biomarker for cancer diagnostics. Since the completion of the human genome project, the demand for cheaper and faster sequencing methods has increased greatly. NGS platforms perform massively parallel sequencing of DNA, during which millions of fragments of DNA from samples are sequenced in unison. The main components of NGS are DNA ligation, DNA library construction, clonal template amplification, and massively parallel sequencing. NGS systems can be characterized on the basis of the sequencing method involved, e.g., pyrosequencing for Roche 454, sequencing by synthesis for Illumina, ion semiconductor-based sequencing for Ion-torrent, or nanopore sequencing for Oxford nano [46].

In this section, we review the representative platforms that have been used for detection of cancer mutations in ctDNA isolated from patients with NSCLC, as summarized in Table 5. Illumina platforms are the most widely used NGS systems worldwide. Among the various products, MiSeq, released in 2011, is a low-throughput, fast-turnaround platform that is best suited for research purposes at smaller laboratories [47]. Target mutations (19 del, L858R, T790M) from the plasma and urine samples of lung cancer patients can be detected successfully using MiSeq [48]. Ion-torrent, from ThermoFisher Scientific, was developed using a microfluidic chip and ion sensors to detect the proton that is released when a nucleotide is incorporated into the polymerase in DNA molecules [47]. It has extremely short run times and short read lengths and does not have bulky settings. These are advantages in detecting target mutations from short-fragmented ctDNA. This platform provides unique panels of clinically relevant hotspot somatic mutations in cancers, and these platforms have been used to screen for cancer effectively by detecting genetic aberrations in the ctDNA of metastatic lung cancer patients [49]. Although the system itself was developed to be easy to use, the level of analytical performance (sensitivity, specificity) is moderate. Therefore, many researchers have attempted to increase the sensitivity and specificity of aspects of the sequencing technology to improve the early detection of cancer.

Tagged-amplicon deep sequencing (Tam-Seq) has been used to screen the 5995 genomic bases for low-frequency mutations, such as ctDNA [50]. This technique employs short amplicons, two-step amplification, sample barcodes, and high-throughput PCR to achieve effective amplification of small amounts of fragmented ctDNA, deduction of false positives stemming from PCR errors, and fast turnaround times (TAT), respectively. Using this method, cancer mutations in ctDNA have been identified at sensitivity and specificity levels >97%.

Cancer-personalized profiling by deep sequencing (CAPP-Seq), designed to address multiple classes of somatic alterations, has been used to identify mutations in >95% of tumors [51]. This approach combines optimized library preparation methods for low DNA input with a multiphase bioinformatics approach to design a “selector” that targets recurrently mutated regions in the cancer of interest. The selector has the ability to identify an individual patient’s cancer-specific genetic aberrations against non-targeted aberrations. This technology has been used to detect ctDNA at an early stage of NSCLC (50% of patients with stage I) with 96% specificity for mutant allele fractions down to ~0.02%. CAPP-Seq has been improved by combining it with pre-specified barcodes and “background polishing” achieved by modeling position-specific errors, which is referred to as integrated digital error suppression (iDES) [52]. iDES-enhanced CAPP-Seq has been shown to be able to accurately detect tumor-derived DNA down to 0.0025%, which is approximately one tenth of the detection limit of the original CAPP-seq system (0.02%). In one study, iDES-enhanced CAPP-Seq was used successfully to analyze the significantly detectable ctDNA in 93% of patients, including three with tumors in early stages (stage I).

A novel approach called targeted error correction sequencing (TEC-seq) was recently developed for ultrasensitive direct evaluation of sequence changes using massively parallel sequencing, which enables the detection of cancer-specific genetic aberrations in ctDNA in early-stage disease [53]. This approach is based on the targeted capture of multiple regions of a genome by optimizing the process of library generation, in combination with pre-specified barcodes. In one study, 200 patients with early stages of cancer (stage I and II) were evaluated to detect somatic mutations in ctDNA from plasma. Of the 200 patients, 59% of 61 patients with stage I or II lung cancer had significantly detectable somatic alterations in ctDNA.

Multi-region exome sequencing (M-seq)-derived tumor phylogenetic trees were developed to conduct ctDNA profiling in early-stage NSCLC [57]. Using this technique, 48% of 96 early-stage NSCLC patients were identified as ctDNA-positive with at least two single-nucleotide variants (SNVs).

In summary, the major advantages of NGS over other detection techniques are the capability to discover new genetic mutations and ultra-sensitive detection. For sensitive detection of known mutations, PCR-based assays, such as BEAMing (beads, emulsions, amplification and magnetics) and ddPCR, have been used to evaluate ctDNA as a screening marker for early diagnosis of cancer. BEAMing is a type of emulsion PCR that incorporates compartmented individual beads with particular DNA molecules [58]. BEAMing is a highly sensitive method that enables the detection of small amounts of somatic mutations in ctDNA. In a clinical study to screen for cancer in NSCLC patients who had taken third-generation EGFR TKI therapeutics (AZD9291, osimertinib), BEAMing was used to detect EGFR mutations from ctDNA with a low limit of detection (0.02% of the allele fraction) [54]. This study also demonstrated the analysis of the cobas^®^ EGFR Mutation Test v2, which is a PCR-based assay that has been developed for the detection of EGFR mutations, including a T790M mutation, for treatment with osimertinib, which was approved by the FDA in 2016 [54].

One of the most powerful detection techniques for screening for cancer based on identification of genetic alterations in ctDNA is ddPCR [59]. A large number of droplets are generated using a microfluidic chip that can be used to compartment particular DNA fragments to greatly increase sensitivity and selectivity, especially for rare target mutations. The first prospective study to demonstrate the utility of ddPCR-based plasma genotyping in advanced NSCLC patients was reported in 2017. In this study, both the EGFR exon 19 del and L858R mutations were detected with 100% specificity, whereas T790M was detected with a lower specificity of 79%, from ctDNA samples from advanced non-squamous NSCLC patients [55]. The PCR-based assays summarized above are expected to be actively utilized in clinical applications, but it remains essential to discover more novel biomarkers using NGS for more effective cancer screening.

### 5.2. Selection of Therapeutic Targets, Monitoring Response, and Prediction of Resistance to Therapy

ctDNA can be used in NSCLC treatment management through the selection of specific therapeutics and monitoring the response to treatment and development of resistance by means of detection of somatic mutations in the EGFR gene. In fact, patients who receive EGFR-TKI treatment usually acquire EGFR-TKI resistance, such as T790M mutations. In a case study, T790M mutations in ctDNA were detected in the plasma of 47% of 117 NSCLC patients using ddPCR analysis [60]. Among patients receiving second-line or later TKI treatment, those who were T790M ctDNA-positive NSCLC had significantly shorter OS than the negative group (median OS: 26.9 months vs. NA). In another study involving 41 advanced NSCLC patients enrolled in a phase II clinical trial of erlotinib and pertuzumab, EGFR mutation status in ctDNA was associated with longer PFS (~7 months vs. NA, *p* = 0.039) [30]. CAPP-Seq was employed to study resistance mechanisms by means of ctDNA analysis of 43 NSCLC patients treated with rociletinib, which is another type of third-generation TKI drug [51]. This study detected multiple resistance mechanisms in 46% of patients after treatment with first-line inhibitors, indicating frequent intra-patient heterogeneity. In another case study, T790M was detected in 31% of plasma samples from 58 patients with T790M negative tumor tissues [61].

The other important advantage of using ctDNA assays is relatively easy serial sampling [55]. In one study, the TAT for liquid biopsy was two days for newly diagnosed NSCLC and three days for acquired resistance, whereas the TAT for tissue genotyping in patients with newly diagnosed NSCLC was significantly longer at 12 days (*p* < 0.001) and 27 days for acquired resistance to EGFR TKI therapy in a real-world clinical setting (*p* < 0.001). The possibility of frequent monitoring of disease progress using ctDNA would be of tremendous benefit, particularly to patients who have developed acquired resistance to the first-line therapy with painful bone metastases but are not able to receive further systemic therapy (because of the limited genotyping data that can be obtained, given the risks of tissue biopsy, depending on the patient’s age and comorbidities). In short, the use of ctDNA analysis in cancer treatment monitoring for NSCLC patients can yield valuable results with high clinical relevance, including investigation of mutation status and serial genotyping of tumors.

## 6. Conclusions and Perspectives

Liquid biopsy, a noninvasive alternative to tissue biopsy, is attracting tremendous interest among cancer clinics because it provides specific information on circulating biomarkers in patients’ blood and helps doctors to design proper treatments for patients at various stages of their disease. Several devices have been developed to isolate CTCs and study their clinical utility as diagnostic, prognostic, and treatment monitoring markers in cancer patients. Similarly, standardized isolation methods, such as solid-phase extraction techniques, have been used to isolate ctDNA, and many sequencing or mutation analysis methods have been developed to study and understand their clinical implications.

In addition, it has been shown that complementary multi-marker analyses including both CTC and ctDNA produces better clinical results than analysis of single circulating biomarkers [38,62]. A study on complementary detection of EGFR T790M mutations based on analysis of both CTCs and ctDNA in 37 advanced NSCLC patients resulted in successful genotyping in 100% of cases [38]. In another study, CTCs and ctDNA of EGFR-mutant NSCLC patients who enrolled in a phase II trial of erlotinib, resulted in 100% detection of T790M mutation in the ctDNA samples of the patients with detected mutations in their tissue samples [62]. Furthermore, in the study cohort, ctDNA was more strongly correlated to progression-free survival than CTCs. However, it is difficult to conclude from this that ctDNA is a better prognostic marker than CTCs because the CTC isolation method used in that study was not a standardized/recommended method for lung cancer patients.

Despite many studies having demonstrated the viability of liquid biopsy using CTC and ctDNA, there is currently no consensus on the criteria for use of these circulating biomarkers as prognostic markers in NSCLC. Among the limitations are lack of congruence; difficulties in standardization of sample types and preparation methods, selection of the isolation method, sample heterogeneity, and variations in marker expression (for example, CTCs that have undergone EMT lack epithelial marker expression), reproducibility, selectivity, and sensitivity. Furthermore, technical issues related to the selection of blood collection tubes, blood volume, and the type of downstream analysis could influence the quality of the data and may contribute to inaccuracies and statistical errors.

The results of a recent liquid biopsy validation study in which identical samples were submitted to two different laboratories for independent testing showed completely matched mutations in only 7.5% of cases. These results underscore the necessity of standardization and robust system development and the importance of multicenter and large-scale clinical validation of each technique before it can be applied to routine clinical use. In addition, standardized protocols are required for blood collection and sampling to minimize technical errors [63].

The ability to apply liquid biopsy to routine clinical use is certain to increase in the future. Previous reports have shown that incorporating complementary multi-marker analyses in liquid biopsy contributes to better understanding of cancer heterogeneity and longitudinal surveillance of cancer therapy. However, to overcome existing limitations, it is necessary to develop robust technologies for isolation and analyses of circulating biomarkers. In addition to blood-based circulating biomarkers, such as CTCs and ctDNA, other circulating biomarkers, such as extracellular vesicles, which are present in almost all body fluids, including blood, have been shown to contain tumor-derived genetic material. These are being studied extensively to assess their clinical utility in diagnosis, prognosis, and treatment monitoring. However, the results need to be validated with independent tests at multiple centers using large cohorts of patients.

More information on the relationships among different liquid biopsy markers will contribute to better understanding of cancer progression and metastasis mechanisms. Complementary use of multi-markers may be beneficial in improving diagnostic, prognostic, and predictive accuracy and achieving the sensitivity and specificity that is required for clinical applications. Active collaborations between clinics and academic research laboratories are necessary for clinical validation of new technologies for liquid biopsy.

## Figures and Tables

**Table 1:** Pros and Cons of tissue biopsy and liquid biopsy.

Tissue Biopsy	Liquid Biopsy
Clinically validated Invasive and risky Difficult to repeat Failure to reflect tumor heterogeneity Failure to detect metastasis at distant sites Impractical for periodic monitoring of treatment response	Clinical practice rules are not yet established Non-invasive Easily repeated Potential to reveal spatial and temporal tumor heterogeneity Offers a more comprehensive picture of the disease Real-time monitoring for drug response and resistance

**Table 2:** Unique advantages and limitations of circulating biomarkers; CTCs vs. ctDNA.

	CTC	ctDNA
**Advantages**	Morphological and functional analysis of cells (ICC ^1^, FISH ^2^) Proteomics at both cellular and sub-cellular levels Analysis of RNA expression In vitro/in vivo functional studies (PDX ^3^ model) Relevance to metastasis	Potential for full representation of spatial and temporal tumor heterogeneity More sensitive to tumor burden, treatment monitoring, and development of resistance First FDA-approved liquid biopsy test for NSCLC Relatively simple isolation method
**Limitations**	Extreme rarity and fragility Heterogeneity of CTCs Lack of standard isolation method Requires better sensitivity and specificity (EMT ^4^, WBC ^5^)	Rarity and fragility Difficult to isolate tumor-specific DNA Contamination with DNA from normal cells No functional analysis

^1^ ICC (Immunocytochemistry); ^2^ FISH (Fluorescent in situ hybridization); ^3^ PDX (Patient-derived xenograft); ^4^ EMT (Epithelial to mesenchymal transition); ^5^ WBC (White blood cells).

**Table 3:** Examples of studies on CTCs-based prognosis for patients with non-small cell lung cancer (NSCLC).

Methods	Therapeutics	Stage	# of Patients	Cut off (CTCs/7.5 mL)	Significance *	Detection Rate
CellSearch	Platinum	IIIA–IV	101	5	PFS/OS (*p* < 0.001)	14.9% [29]
	EGFR TKI	IIIB–IV	37 ^a^ (41) ^b^	1	PFS (*p* = 0.006) **	75.7% [30]
	EGFR TKI	IIIA–IV	59	2	PFS/OS (*p* = 0.01/*p* = 0.006)	40.7% [31]
	QT treatment	IIIB–IV	43	5	PFS/OS (*p* = 0.034/*p* = 0.008)	23.2% [32]
	Platinum, EGFR TKI, ALK inhibitor	IIIB–IV	125	5	OS (*p* = 0.022)	19.2% [33]
	Adjuvant chemotherapy	I–IIIA	27 ^a^ (30) ^b^	1	DFS/OS (*p* = 0.011/*p* = 0.037)	22.2% [34]
ISET	Neoadjuvant therapy	I–IV	208	50 ***	DFS/OS (*p* = 0.001/*p* = 0.002)	30.8% [35]
	Neoadjuvant therapy/Surgery	I–IV	210	1	DFS (*p* < 0.0001)	49.5% [36]

* Progression-free survival (PFS), overall survival (OS), disease-free survival (DFS). *p* values in [31] and [33] were determined from multivariate Cox-proportional hazards regression analysis. *p* values in the other references were determined by Kaplan–Meier analysis; ** Determined from CTC count change 56 days after treatment (baseline CTC: not available); *** This CTC count is the number of CTCs in 6 mL of blood, not normalized to 7.5 mL. ^a^ number of patients whose blood samples were analyzed; ^b^ number of patients who enrolled in the study.

**Table 4:** Summary of reports on mutation detection in CTCs from patients with NSCLC.

Isolation Method	Capture Efficiency	Through-Put (mL/h)	Purity **	# of Patients	Detection Techniques	Mutation	Detection Rate	Con-Cordance
^Hb^CTC-Chip [37,38]	91.8% ± 5.2%	1.2	14.0% ± 0.1%	28 ^a^ (40) ^b^	DNA sequencing	T790M	50% (14/28)	57% (12/21)
Nano velcro [39]	>70%	0.5	> 35% (4350 WBCs → 12 WBCs) ***	7	Sanger sequencing	L858R/T790M	85.7% (6/7) 28.6% (2/7)	100% (7/7)
Single cell retrieval [40]	>95%	3.0	100%	6 ^a^ (7) ^b^	Sanger sequencing	L858R/T790M	16.7% (1/6) 66.7% (4/6)	100% (6/6)
MagSifter [41,42]	95.7% *	10	17.7% ± 9.3% (368 ± 299 WBCs/mL)	7 ^a^ (35) ^b^	RT-PCR	19 del/L858R/T790M	42.9% (3/7) 14.3% (1/7) 42.9% (3/7)	NA
OncoBean Chip [43,44]	>80%	10	390–740 WBCs/mL	4 ^a^ (36) ^b^	RT-PCR	EGFR/KRAS	25.0% (1/4) 50.0% (2/4)	NA

^a^ Number of patients whose CTCs were isolated and used for genotyping; ^b^ number of patients who enrolled in the study. Nanovelcro and Single cell retrieval required at least 3 repeated processes. * Capture efficiency of MagSifter >95%, release efficiency 92.7 ± 6.1%, cells collected in eluted fraction 89.6 ± 12.1%; ** Purity was measured by spiked experiment except MagSifter (clinical sample experiment); *** After 2nd round of purification, number of WBCs decreased from 4350 WBCs/mL to 12 WBCs/mL.

**Table 5:** Summary of reports on mutation detection in ctDNA from patients with NSCLC.

Detection Techniques		Sensitivity	Specificity	Detection Target	Detection Limit (Threshold)
**NGS**	MiSeq [48]	93% (T970M), 100% (L858R), 87% (19del)	94% (T970M), 100% (L858R), 96% (19del)	T790M, L858R, 19del	0.028%
	Ion Torrent [49]	58%	87%	Panel: 50 genes	0.2%
	TAM-seq [50]	97%	97%	6 genes *	2%
	iDES-CAPP-seq [52]	90%	96%	Panel: 292 genes	0.0025%
	TEC-seq [53]	97.4%	>99.9999%	Panel: 55 genes	0.05%
**PCR-based assay**	BEAMing [54]	81% (T970M), 87% (L858R), 82% (19del)	58% (T970M), 97% (L858R), 97% (19del)	T790M, L858R, 19del	0.02%
	Cobas EGFR Mutation Test [54]	73% (T970M), 87% (L858R), 82% (19del)	67% (T970M), 97% (L858R), 97% (19del)	T790M, L858R, 19del	0.02%
	ddPCR [55]	77% (T970M), 69% (L858R), 86% (19del)	63% (T970M), 100% (L858R), 100% (19del)	T790M, L858R, 19del, KRAS G12X	NA/0.04% **

* PIK3CA, EGFR, BRAF, PTEN, KRAS, TP53; NA—not available; ** Ref. [56].
